# Ecosystem Coupling and Ecosystem Multifunctionality May Evaluate the Plant Succession Induced by Grazing in Alpine Meadow

**DOI:** 10.3389/fpls.2022.839920

**Published:** 2022-03-04

**Authors:** Yingxin Wang, Zhe Wu, Zhaofeng Wang, Shenghua Chang, Yongqiang Qian, Jianmin Chu, Zhiqing Jia, Qingping Zhou, Fujiang Hou

**Affiliations:** ^1^State Key Laboratory of Grassland Agro-Ecosystems, College of Pastoral Agriculture Science and Technology, Lanzhou University, Lanzhou, China; ^2^Key Laboratory of Grassland Livestock Industry Innovation, Ministry of Agriculture and Rural Affairs, College of Pastoral Agriculture Science and Technology, Lanzhou University, Lanzhou, China; ^3^Grassland Research Center of National Forestry and Grassland Administration, Research Institute of Ecological Protection and Restoration, Chinese Academy of Forestry, Beijing, China; ^4^Research Institute of Forestry, Chinese Academy of Forestry, Beijing, China; ^5^College of Qinghai Tibet Plateau Research, Southwest Minzu University, Chengdu, China

**Keywords:** plant succession, ecosystem coupling, ecosystem multifunctionality, grazing, alpine meadow

## Abstract

Most alpine meadow on the Tibetan Plateau are at different stages of community succession induced by grazing practices. Quantifying the succession sequence and assessing the dynamics of plant composition, ecosystem coupling, and multifunctionality across successional stages are essential for reasonable restoration of degraded alpine meadow. Here, we selected areas with different grazing disturbance histories and used them as a space series (i.e., space-for-time substitution) to study the community succession. Our work quantified the plant succession sequence of alpine meadow induced by grazing with plant functional group approach. The plant succession sequence is from the tall sedge community with erect growth to the short undesirable toxic forbs community with prostrate growth. Ecosystem coupling, ecosystem multifunctionality and their relationships were all the lowest in Stage 4. Compared to Stage 4, the ecosystem multifunctionality index increased in Stages 1, 2, and 3 by 102.6, 89.8, and 207.6%, respectively; the extent of ecosystem coupling increased by 20.0, 16.8, and 21.2%, respectively. Our results indicated that the driving factors of ecosystem coupling and ecosystem multifunctionality were soil factor individual in early successional stage to plant-soil simultaneously in late successional stage. Our results also highlighted the importance of toxic weeds during the late stage of degraded succession and suggest that the expansion of toxic plants is a consequence of their greater suitability from a successional perspective. The findings of this study would provide valuable guidance for optimizing the management and restoration practice of alpine meadow.

## Introduction

Plant community succession is the process of species replacement (Lavorel and Garnier, 2010; Milchunas and Vandever, 2013) or the change in plant functional traits over time (Kahmen and Poschlod, 2004; He et al., 2019). Facilitation, inhibition, and tolerance are the three main different turnovers between species in the succession pathways (Maggi et al., 2011; Zhang and Tielbrger, 2019). Biological mechanisms that underlie succession events include competition colonization trade-off, successional niche mechanisms, facilitation, interactions with enemies, and the resource-ratio hypothesis (Rodríguez et al., 2007; Muscarella et al., 2016; Prugh and Sivy, 2020). Furthermore, three successional processes were dissected which included site availability, differential species availability, and differential species manifestation (Baeten et al., 2010). Specifically, the mechanisms within each process are driven by biotic and abiotic factors and their interactions, which are further regulated through such factors as climate change, fire, and livestock grazing (Nicolai, 2019; Leizeaga et al., 2020).

The effect of livestock grazing on the differential performance of plants is important in successional process (Milchunas and Vandever, 2013; Howard et al., 2020). In addition to directly foraging and ingesting the shoot of plants and changing the competitive balance among plant species (Duhamel et al., 2019), livestock grazing can also influence successional rates and successional trajectories by impacting seed dispersal and colonization patterns (Wang et al., 2018; Ma et al., 2020). Grazing can cause “arrested successional development” that results from preferential feeding on late successional species (Kemp and King, 2001). Selective feeding of livestock on early successional species can promote the emergence and growth of late successional species, thereby accelerating succession (Devaney et al., 2020). Meanwhile, diversified livestock assemblage has additive or complementary effects on successional trends (Wang et al., 2019). For example, cold-season grazing could encourage alpine grassland succession from a sedge/forb community to a grass/forb community because yak and Tibetan sheep prefer to consume sedge plants (Shi et al., 2019; Sun et al., 2020). Also, seedling herbivory and defense play a fundamental role in regulating the successional trajectories by livestock grazing (Hanley and Lamont, 2001). At this period, particularly, plants are easily vulnerable to organs loss in their life cycle (Hanley and Lamont, 2001). Hence, herbivory can decrease seedling vigor, further to affect the competitive ability and opportunity of long-term survival, even lead to mortality (Bestelmeyer et al., 2010; Barton and Hanley, 2013). A 6-fold reduction in plant seedling survivorship resulted from feeding by large herbivores in Panamanian forests (Asquith et al., 1997). The mechanism behind them is likely to the interspecific difference in seedling tastiness, size, and morphology, also from differences in species richness, pattern of spatial distribution, and seedling emergence occasion (Lusk and Kelly, 2003). Further, seedling establishment can be favored from microsite availability promotion via physical disturbances of livestock, and from creating more chances for seedling recruitment by reducing litter loads and increasing availability of light (Marcora et al., 2013; Ameztegui and Lluí, 2015). Additionally, change in plant species composition influencing energy and nutrient flow and ecosystem succession by altering litter decomposition (Song et al., 2017; Barnes et al., 2018).

Evaluation of dynamics of plant and soil properties can offer more details on ecosystem stability and sustainability, thereby contribute to predict the successional trajectory induced by grazing practices (Tang et al., 2010; Paterno et al., 2016; Shi et al., 2018).

In China, researches related to the classification of the degradation succession of grazed grasslands (e.g., a typical steppe (Zhao et al., 2017), desert steppe (Hu et al., 2016) have long been recognized, but relatively little is known about the degradation succession of alpine meadow. Increased grazing pressure and climate change accelerated grassland degradation, leading to dysfunction of the alpine meadow ecosystem. Overgrazing may also decrease sward height, vegetation cover, and biomass and may increase undesirable and unpalatable grass species and even the occurrence of species that are toxic to animals (Wu et al., 2015). Besides, a reduction litter decomposition to the soil, which results from the degradation of vegetation, can lead to a substantial decrease in amination, nitrification, and nitrogen fixation, resulting in a rapid decrease in soil fertility (Dong et al., 2015; Howard et al., 2020). These effects, in turn, reduce the flow of energy and substance circulation in the ecosystem, finally resulting in discordance between the two dominant components (i.e., soil and plant), as well as in the disruption of ecosystem multifunctionality (Wang et al., 2020a; Zhang R. Y. et al., 2021).

More tightly coupled ecosystems can in favor of more extensive ecosystem functions, which are likely to be associated with a higher efficiency in resources utilization and material cycle (Risch et al., 2018). However, a dearth of information on this topic on effects of succession stages induced by grazing on ecosystem coupling and multiple ecosystem functions. Additionally, we have little knowledge about how these bidirectional interactions and relationships may vary throughout the successional process, and their underlying mechanisms. To address these knowledge issues, we used plant functional groups instead of plant species to identify and quantify the stages of succession. We also have addressed the following questions: (1) Are there substantial differences in plant composition, ecosystem coupling, ecosystem multifunctionality, and the relationships among these functions during the different successional stages? If yes, (2) What the main driving mechanism in difference of ecosystem coupling and ecosystem multifunctionality affected by plant succession? We assumed that (1) plant composition, ecosystem coupling, ecosystem multifunctionality and their relationships would change in different successional stages? (2) the environmental factors and driving mechanism associated with ecosystem coupling, ecosystem multifunctionality would differ among successional stages induced by grazing. The results of this study provide valuable guidance for optimizing the management and restoration practice of alpine meadow.

## Materials and Methods

### Study Site

The experiment was carried on at the Institute of Tibetan Plateau at Southwest Minzu University (32°48'N, 102°33'E, 3,500 m above mean sea level), located about 4 km north of Hongyuan County, Sichuan, China. The mean annual temperature and precipitation is 1.1°C and 700 mm, respectively (Liu et al., 2020). The coldest month is January (~9.7°C) and the warmest month is July (11.1°C). The majority of rainfall is concentrated in warm season (June to September). The soil type is alpine meadow soil (Chinese Soil Taxonomy Research Group, 1995). The type of vegetation is classified as alpine meadow and comprises sedges (most frequently *Kobresia cristata, Scirpus pumilus*), grasses (most frequently *Elymus nutans, Poa pratensis*, and *Agrostis* species), and forbs (dominated by *Saussurea* species and *Anemone* species). The study site has been used for trial plots under different land use patterns caused by different grazing practices since 2007.

### Field Methods

There are various vegetation structure and composition in the experimental areas that have different grazing disturbance histories. The common approach referred to as space-for-time substitution (Pickett, 1989) was used to infer successional patterns of alpine meadow. The advantage of this approach is that plant community variation can be investigated well without long-term observations of a single plot, which need take decades to finish (Li et al., 2015). The belt transect method was used to measure vegetation in the experimental area in August, 2018. We randomly set 10 belt transects with a length of 200 m each. Three quadrats (0.5 × 0.5 m) were randomly selected in every 10 m of each sample strip. In total, there were ~600 quadrats (samples).

### Data Collection

#### Sampling of Plant Properties

To investigate the community structure and species composition of each quadrat, the height of the tallest stem of each plant species was recorded. Total number of plant species of each quadrat represent plant species richness (SR). All on-ground plants were removed and bagged to bring back to the lab. Then, we separated them for each species and weighed to obtain aboveground biomass (AGB) after oven-dried at 65°C for 48 h.

#### Sampling of Soil Properties

The surface layer's (a depth of 10 cm) soil temperature (ST) and soil water content (SM) of each quadrat were measured using Field-Scout TDR-100 (Spectrum Technologies, Plainfield, IL, USA). Cutting ring method was used to measure Soil bulk density (SBD). The soil was sampled in the middle of each 0.5 × 0.5 m quadrat using a soil auger, and then placed them in mesh bags (2-mm mesh). After being air-dried about a month outdoors, the soil samples were separated root and soil sub-samples. The root sub-sample was washed free of soil and weighed to acquire belowground biomass (BGB) in the following being oven-dried at 115°C for 48 h.

The soil sub-samples were further to air-dried in laboratory at room temperature and were sieved through a 0.2-mm mesh. An Element Analyzer was used to determine soil total carbon (STC), soil total nitrogen (STN), and soil total sulfur (STS). Soil total phosphorus (STP) was measured using the molybdate colorimetric test after perchloric acid digestion.

### Ecosystem Characteristic Calculations

#### Plant Species Evenness Index

The Camargo evenness index (EV) is calculated independently of SR (Camargo, 1993), and is defined as formula 1:


(1) $$\begin{array}{l} E=1-\overset{s}{\underset{i}{\sum }}\overset{s}{\underset{j=i+1}{\sum }}\left(|P_{i}-P_{j}|/S\right) \end{array}$$

where *E* is the Camargo evenness index, *P*_*i*_ is the biomass proportion of plant species *i* in the sample, *P*_*j*_ is the biomass proportion of plant species *j* in the sample, and *S* is the total number of plant species of the quadrat.

#### Ecosystem Coupling Between Soil and Plant

The indicator of coupling coordination degree between plant and soil subsystems can be used for evaluating the ecosystem coupling between soil and plant (EC_SP). To eliminate the influence of dimension and magnitude, we first standardized the raw data using two formulas (Tang, 2015; Wang et al., 2020a).

For the positive index, we used formula 2:


(2) $$\begin{array}{l} {X^{\prime }}_{i}=\frac{X_{i}-minX_{i}}{\max\;X_{i}-minX_{i}} \end{array}$$

For a negative index, we used formula 3:


(3) $$\begin{array}{l} {X^{\prime }}_{i}=\frac{\max\;X_{i}-X_{i}}{\max\;X_{i}-minX_{i}} \end{array}$$

where, *X*_*i*_ represent the original value of index *i, X'*_*i*_ represent the standardized value of index *i*, and *maxX*_*i*_ and *minX*_*i*_indicate the maximum and minimum value of the index *i*.

To evaluate the soil and plant subsystem, we supposed *X*_1_, *X*_2_, …, *X*_*i*_ to stand for soil subsystem indexes (*X*_*s*_), and *Y*_1_, *Y*_2_, …, *Y*_*j*_ to stand for plant subsystem indexes (*Y*_*p*_). Then calculated by formula 4 and 5:


(4) $$\begin{array}{l} S\left(X\right)=\overset{i}{\underset{s=1}{\sum }}W_{s}X_{s}^{\prime } \end{array}$$

(5) $$\begin{array}{l} P\left(Y\right)=\overset{j}{\underset{p=1}{\sum }}W_{p}Y_{p}^{\prime } \end{array}$$

where *S(X*) and *P*(*Y*) are the integral value of the soil and plant subsystem, respectively; *X'*_*s*_ and *Y'*_*p*_ are the standardized values of *X*_*s*_ and *Y*_*p*_, respectively, and *W*_*s*_ and *W*_*p*_ are the weight of *S*(*X*) and *P*(*Y*), respectively, which can be calculated by principal component analysis (PCA).

The degree of coupling coordination was calculated as formula 6:


(6) $$D_{sp}=\sqrt{\left(\frac{{\left[S\left(X\right)\times P\left(Y\right)\right]}^{\frac{1}{2}}}{\frac{\left[S\left(X\right)+P\left(Y\right)\right]}{2}}\right)\times [\alpha \times S\left(X\right)+\beta \times P\left(Y\right)}]$$

where *D*_*sp*_ stand for the coupling coordination degree and α and β stand for the contribution from the soil and plants, respectively.

### Ecosystem Functions

To quantify the quantity and value of ecosystem functions, we chose 12 plant and soil variables including AGB, BGB, SR, plant density (PD), plant height (PH), ST, SM, SBD, STC, STN, STP, and STS in the current study. Based on the selected indicators, the plant growth index (PGI), soil carbon accumulation index (SCI), soil nutrient cycling index (SNI), and ecosystem multifunctional index (EMF) was calculated via an average method as formula 7:


(7) $$\begin{array}{l} EMF=\frac{1}{N}\overset{N}{\underset{i=1}{\sum }}f\left(xi\right) \end{array}$$

where *N* is the number of measured functions and equal to 12 in this study, *x*_*i*_ represents the measured value of function *i*, *f*(*xi*) is the standardization of *x*_*i*_ via Z-score standardization.

### Statistical Analysis

Clustering detection (Ward method) was performed to reveal the trend and direction of plant community succession of alpine meadow (Supplementary Figure S1). The data for the vegetation and soil properties of the 30 quadrats associated with each stage of succession were used for data statistical analyses.

All data analyses were performed in R version 4.1.2 (R Core Team, 2021). Data distributions and normality was examined through Shapiro-Wilk goodness-of-fit test. First, general linear model from the “ASREML” package (Gilmour et al., 2015) to test the effects of succession stage on the SR, EV, AGB, BGB, PD, PH, SBD, SM, ST, STC, STN, STP, PGI, SCI, SNI, EC_SP, EMF, and the biomass proportion of toxic plants. Thereafter, Tukey's HSD test in the “AGRICOLAE” package (de Mendiburu, 2014) was conducted to assess the differences among means. Second, correlation analysis was performed using the “GGCOR” package (Huang, 2019) to acquire the relationships among SR, EV, PGI, SCI, SNI, and EMF, along with their relationships to EC_SP (Mantel test). Third, simple regression analysis was used to compute the relationships between EC_SP and EMF at different successional stages, along with their relationships to the biomass proportion of toxic plants. Fourth, gradient regression analysis in “GBM” package was used to analysis the relative importance of biotic and abiotic factors to EC_SP and EMF. In the nonlinear model, Friedman's H-statistic was calculated to evaluate the relative strength of the interaction effect (Li et al., 2021). Finally, heatmap analysis from the “PHEATMAP” to realize correlations between SR, EC, ecosystem function indexes (PGI, SCI, SNI, and EMF) and plant, soil properties. All figures were drawn using Origin 2021b and the “GGPLOT2” package.

## Results

### Quantification of Plant Succession

**Figure 1:**
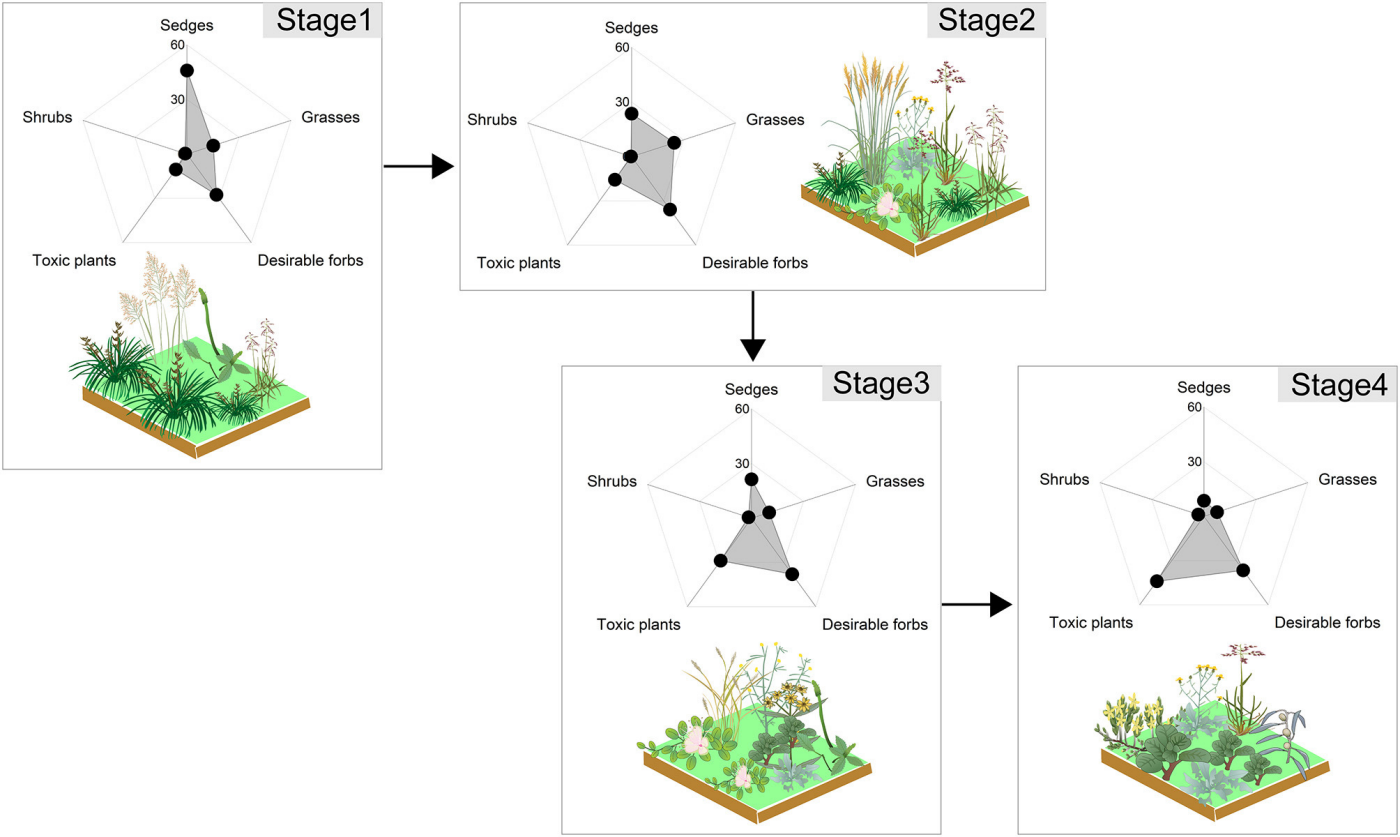
The plant community succession of an alpine meadow of the Tibetan Plateau as a result of grazing. Dominant sedge genera included *Kobresia* and *Carex*; dominant grass genera included *Elymus* and *Poa*; dominant desirable forb genera included *Saussurea* and *Potentilla*; dominant toxic plant genera included *Gentianaceae, Ranunculaceae*, and *Euphorbiaceae*; and dominant shrub species consisted of *Berberis wilsonii* and *Potentilla fruticosa*.

Biomass ratios of plant functional groups was used to quantify the stages of succession (Figure 1). The succession series is as follows: Stage 1 (sedges, 46%; desirable forbs, 28%; grasses, 15%), Stage 2 (desirable forbs, 36%; grasses, 25%; sedges, 24%), Stage 3 (desirable forbs, 38%; undesirable toxic plants, 29%; sedges, 23%), and Stage 4 (undesirable toxic plants, 44%; desirable forbs, 37%; grasses, 11%). From the tall *Kobresia* community with erect growth to the short undesirable toxic forbs community (e.g., *Gentianaceae, Ranunculaceae*) with prostrate growth; the vertical, perennial desirable forbs community represented the transition between these two stages (Figure 1).

### Changes in Characteristics of Plant and Soil Factors Across the Successional Stages

**Figure 2:**
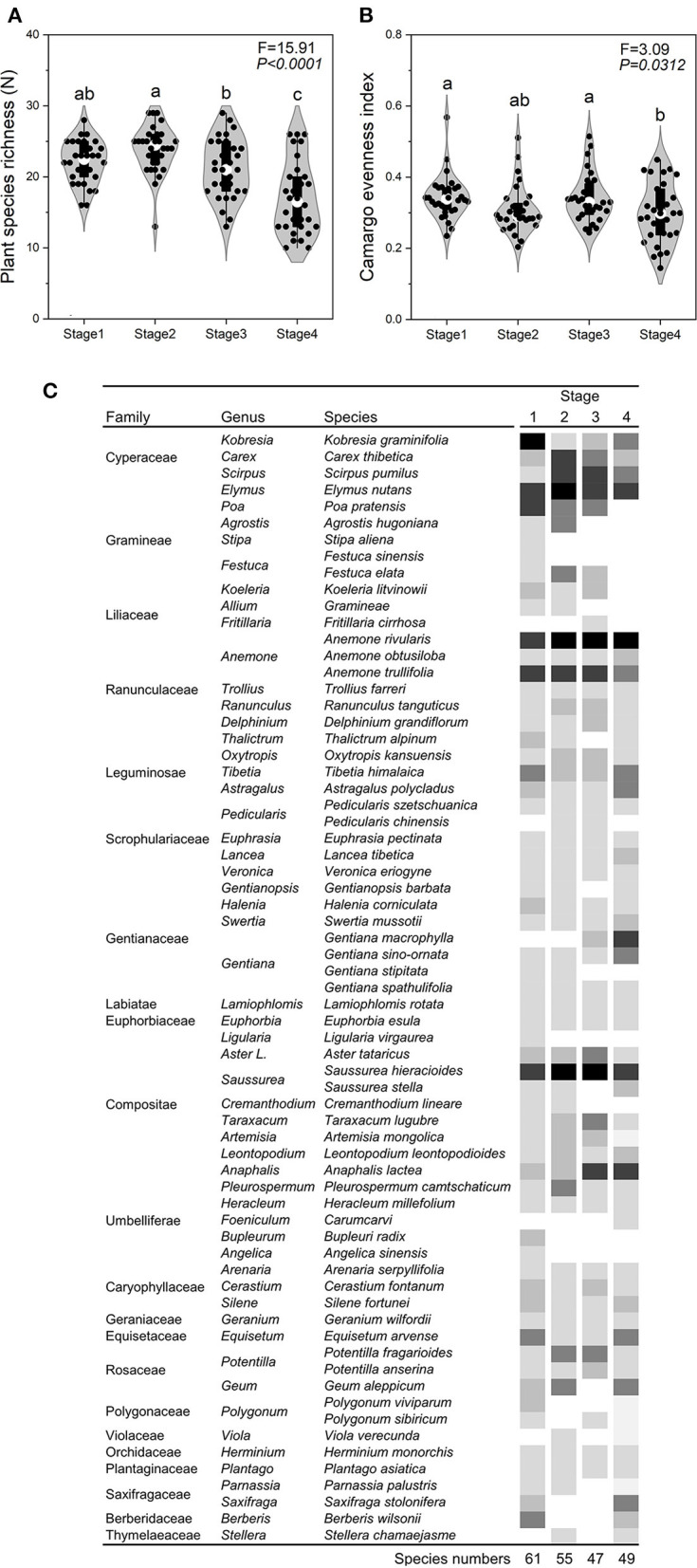
Changes characteristics across the four stages of succession. Plant species richness **(A)**, Camargo evenness index **(B)**, and plant composition **(C)** across the successional stages. In Stage 1, the dominant plant species were *Kobresia graminifolia, Elymus nutans, Poa pratensis, Anemone rivularis*, and *Tibetia himalaica*; in Stage 2, the dominant plant species were *Elymus nutans, Scirpus pumilus, Pleurospermum camtschaticum, Saussurea hieracioides*, and *Potentilla fragarioides*; in Stage 3, the dominant plant species were *Anaphalis lacteal, Saussurea hieracioides, Anemone trullifolia*, and *Aster tataricus*; in Stage 4, the dominant species were *Gentiana macrophylla, Anemone rivularis, Saussurea hieracioides*, and *Oxytropis kansuensis*). The color in **(C)** means the relative biomass of each plant species at different plant successions. The value of relative biomass was higher when the color was darker.

**Figure 3:**
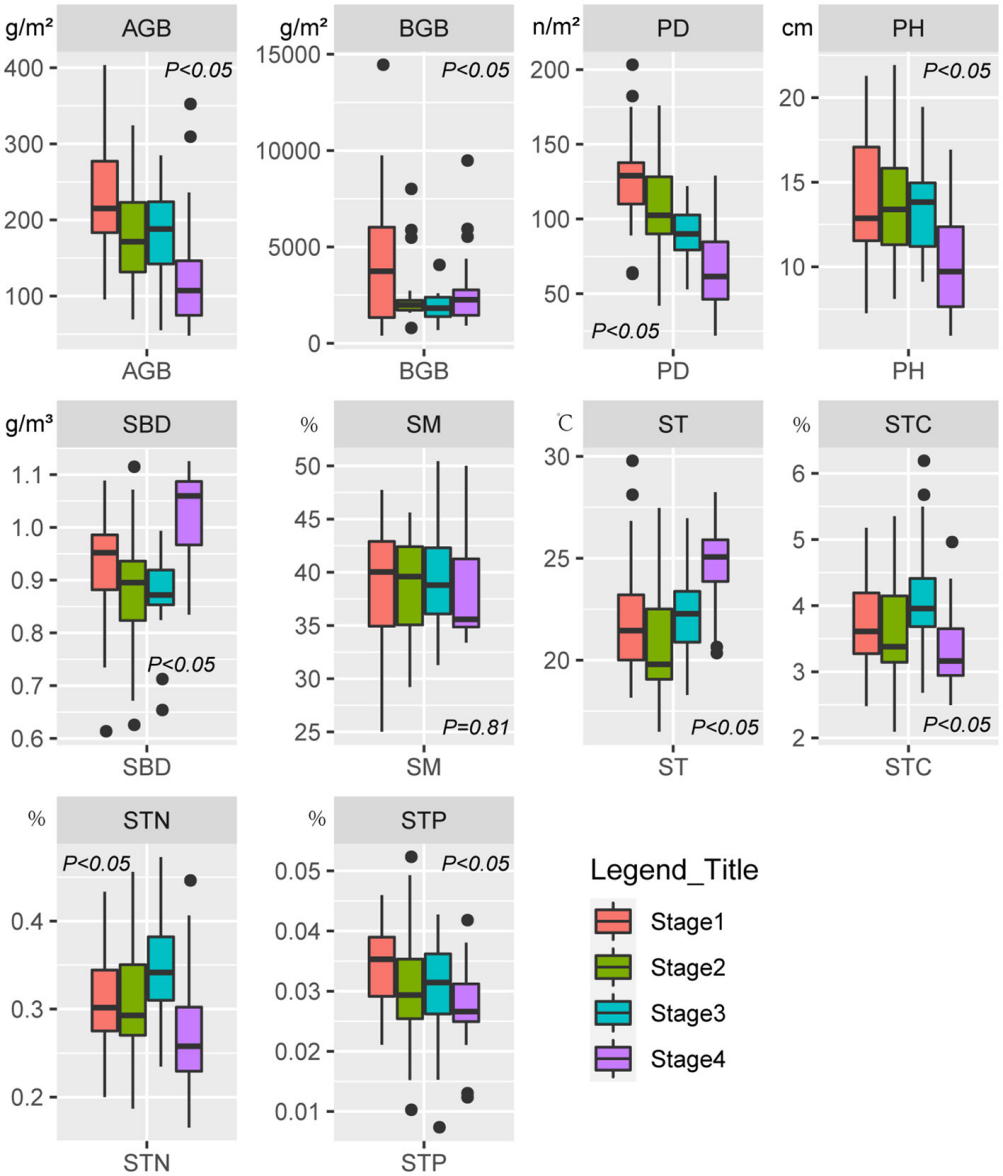
Box plots showing changes in the aboveground biomass (AGB), belowground biomass (BGB), plant density (PD), plant height (PH), soil bulk density (SBD), soil moisture (SM), soil temperature (ST), soil total carbon (STC), soil total nitrogen (STN), and soil total phosphorus (STP) in different successional stages.

The SR, EV, AGB, PD, PH, and STN of Stage 4 was significantly (*P* < *0.05*) lower than that of the other three stages (Figures 2, 3). In contrast, SBD and ST in Stage 4 was significantly (*P* < *0.05*) higher than that of the other three stages (Figure 3). The gamma diversity of each stage (from Stage 1, 2, 3, and 4) was 61, 55, 47, and 49 species, respectively (Figure 2C). There was no significant difference (*P* > *0.05*) in SM among four stages (Figure 3).

### Ecosystem Functions and Ecosystem Coupling and Their Relationships Across the Successional Stages

**Figure 4:**
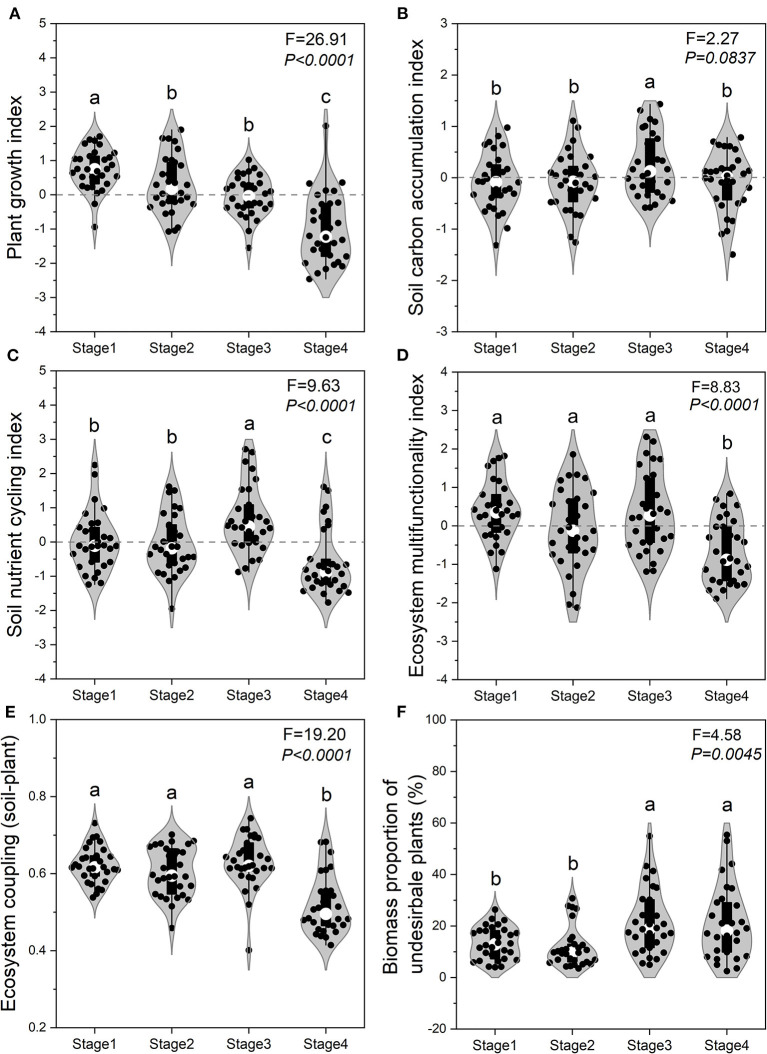
Ecosystem functions across the four stages of succession. Plant growth index **(A)**, soil carbon accumulation index **(B)**, soil nutrient cycling index **(C)**, ecosystem multifunctionality index **(D)**, ecosystem coupling (soil-plant) **(E)**, and biomass proportion of toxic weeds **(F)** across the successional stages. Different lowercase letters above each violin plot indicate statistically significant differences between different succession stages.

The SCI and SNI of Stage 3 were both significantly (*P* < *0.05*) higher than that for the other three stages (Figures 4B,C). The EMF and extent of EC_SP were both at their lowest levels in Stage 4 (Figures 4D,E). Compared with the EMF in Stage 4, this index increased by 102.6, 89.8, and 207.6% in Stage 1, 2, and 3, respectively (Figure 4D). Compared with the extent of EC_SP in Stage 4, there was a 20.0, 16.8, and 21.2% increase in coupling in Stage 1, 2, and 3, respectively (Figure 4E). Biomass proportion of toxic plants in Stage 3 and Stage 4 was significantly (*P* < *0.05*) higher than that in Stage 1 and Stage 2 (Figure 4F).

**Figure 5:**
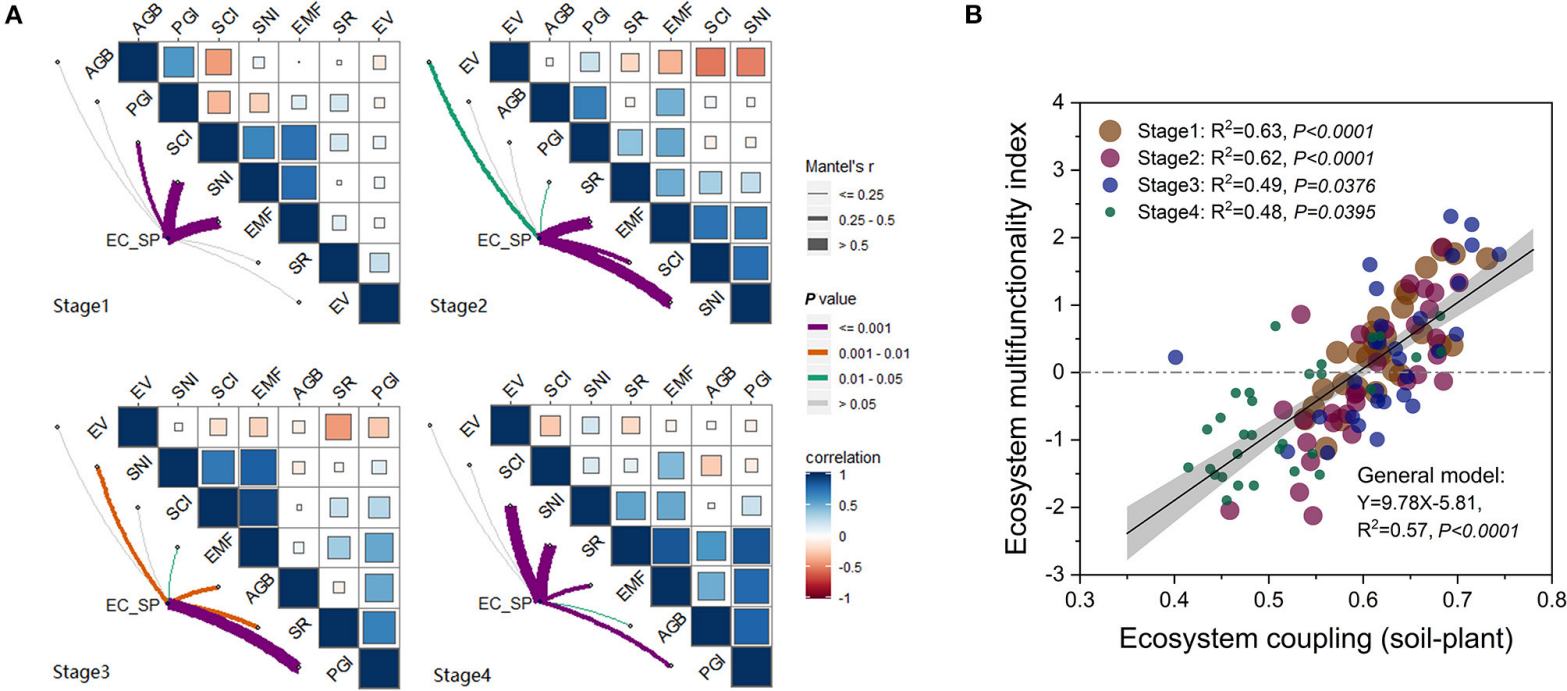
**(A,B)** Correlations between ecosystem coupling and functionality in each successional stage of alpine meadow. AGB, aboveground biomass; SR, plant species richness; EV, Camargo evenness index; PGI, plant growth index; SCI, soil carbon accumulation index; SNI, soil nutrient index; EMF, ecosystem multifunctionality index; EC_SP, extent of ecosystem coupling (soil-plant).

In general, there was a significant positive relationship (*R*^2^ = 0.57, *P* < *0.05*) between the extent of EC_SP and EMF (Figure 5B). Relationships between EC_SP and EMF were at variance in different stages (Figure 5A). Coefficients of correlation between EC_SP and EMF in Stage 1 and Stage 2 were higher than those in Stage 3 and Stage 4 (Figure 5A). EC_SP and EMF were both significantly (*P* < *0.05*) and positively related to SCI and SNI in Stage 1 and 2 and to the soil nutrient index in Stage 4 (Figure 5A). In Stage 3 and Stage 4, EC_SP and EMF both had significant relationships with PGI and SR (Figure 5A).

## Discussion

### Plant Succession Sequence Based on Plant Function Group

Quantification of sequence of community succession is central for raveling out the function and structure of alpine meadow (Bagchi et al., 2013; Avolio et al., 2015). The best way to characterize and identify the process of succession is by analyzing the changes in vegetation composition over the course of a long-term, single-site experiment (Kahmen and Poschlod, 2004; Abbas et al., 2019). However, long-term records of trajectories of community change do not exist, either empirically or experimentally, probably because, at least for plants, the process of succession from pioneer to climax species may take 25–150 years to complete (Porensky et al., 2017). Generally, using a space series to replace a time course is an accepted method for analyzing community succession (Duan et al., 2008; Hu et al., 2016). Plant functional group (PFG) pathway could eliminate the disadvantages of individual plant species approach because of each PFG has its specific and vital role in the grassland ecosystem by altering the stability and functions (Bermejo et al., 2012; Sun and Wang, 2016). Accordingly, we recommend using the biomass proportion of plant functional groups as an effective and integrated index for evaluating the succession trajectory of alpine meadows. Our results demonstrated that the community successional series of alpine meadow were divided into four stages with a trend over time from tall erect-growth sedges community (e.g., *Kobresia*) to short prostrate-growth undesirable toxic forbs community (e.g., *Gentianaceae, Ranunculacese*) (Figure 2). These findings were also consistent with the actual situation determined by experienced observation of the field work (Zhang Z. et al., 2021) and can be viewed as a general degenerate community succession series for alpine meadows for restoration practices. The succession series can contribute to restoration practices in three aspects as following: (i) further clarification of community structure development, (ii) more cognition of species interactions and of nutrient dynamics, and (iii) better understanding of the species transformation between successional stages and of how these stages fit together into successional trajectories (Walker and del Moral, 2009; Zhang et al., 2015).

### Types and Drivers of Plant Composition to Plant Succession

During the process of plant succession induced by grazing, the structure and function of an alpine meadow are affected by changes in the species composition and richness, soil nutrient availability, and a number of abiotic factors including environmental filtering and dispersal mechanisms (Niu et al., 2016). In this study, the changes in across the successional stages can be categorized into the three following types: (i) The decay type is characterized by a decrease in the dominance of a given species or dominance in conjunction with an increase in grazing intensity, as exemplified by *Kobresia graminifolia, Gramineae* species (*Poa pratensis, Stipa aliena*, and *Festuca sinensis*), and *Fritillaria cirrhosa*; (ii) The growth type is characterized by an increase in the dominance of a given species in conjunction with increased grazing intensity. This was noted for *Anemone rivularis, Gentiana macrophylla*, and *Anaphalis lacteal* and (iii) The fluctuating type, which included *Carex thibetica, Scirpus pumilus, Elymus nutans*, and *Potentilla fragarioides*, undergoes changes that are not consistent across the stages (Figure 2).

In a system such as this alpine meadow, the grazing intensity, and selective foraging of the yaks should be the major factors driving change in the species composition (Dong et al., 2015). Rational grazing can promote the regeneration and development of erect bunch grasses (e.g., *Scirpus pumilus, Elymus nutans*, and *Poa pratensis*) by the fragmentation of tussocks, deposition of manure, removal of standing dead plant tissues, and the stimulation of soil water and nutrient transformation and utilization (Sun et al., 2017; Wang et al., 2018). Also, toxic plants and therophyte are easily germinate and become dominant in the open space among the tussocks resulted from livestock trampling or at excrement patches (Zhang et al., 2020), which is consistent with their dominance under heavy grazing practices (i.e., Stage 4 in this study). During early succession stages, there is no selective advantage for livestock to specialize on specific species, for most of them are edible and desirable. Hence, more plenty of rapid growth plant species with low defense chemicals would be dominant owing to the low consumption of resources to herbivory (Wang et al., 2010; Milchunas and Vandever, 2013). In contrast, resource availability is relatively at low level in the late succession. Given these conditions, slow-growing, well-defended toxic weeds should dominate due to the great cost of resources to herbivory (Bosc and Pauw, 2020).

### Driving Mechanism of Ecosystem Coupling and Ecosystem Multifunctionality to Plant Succession

Plant regressive succession weakens the nutrient cycle and energy flow of ecosystem, ultimately leading to discordance between two dominant components (i.e., soil and plant), as well as in maladjustment of ecosystem multifunctionality in alpine meadow (Wang et al., 2020a). Special standards for grassland restoration emphasize the need for comprehensive evaluations (Gann et al., 2019), yet, plant–soil feedback studies in grassland degradation succession remains controversial and how successions stages shape ecosystem multifunction is still unclear. In consequence, using comprehensive indicators such as the extent of ecosystem coupling, ecosystem multifunctionality, and their relationships should provide a broader understanding and sight of the succession process and mechanism induced by grazing.

**Figure 6:**
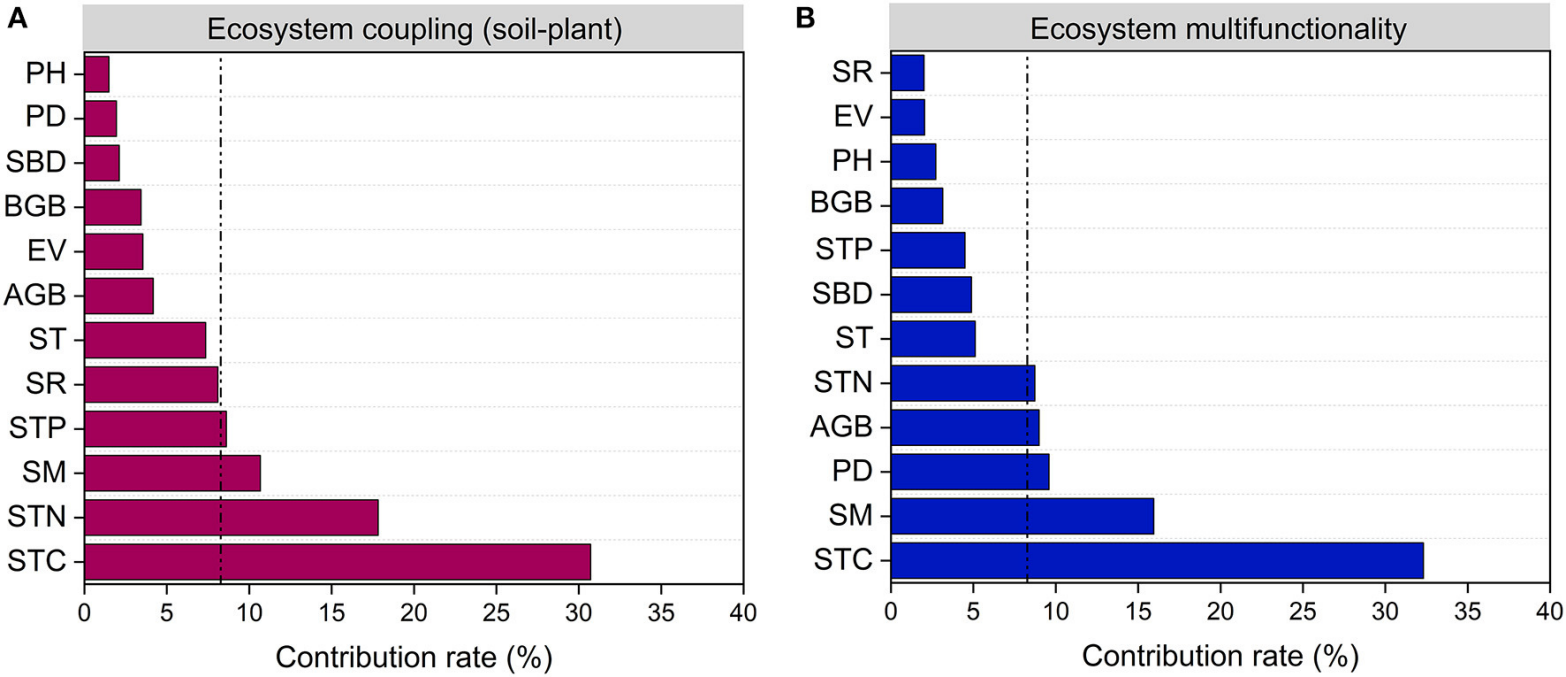
Relative importance of environmental and biological factors explaining ecosystem coupling **(A)** and ecosystem multifunctionality **(B)**. AGB, aboveground biomass; SR, plant species richness; EV, Camargo evenness index; BGB, belowground biomass; PD, plant density; PH, plant height; SBD, soil bulk density; SM, soil moisture; ST, soil temperature; STC, soil total carbon; STN, soil total nitrogen; STP, soil total phosphorus.

The difference in EC_SP, EMF and their relationships may reflect the differences in comprehensive changes of plant succession (Figure 5; Supplementary Figure S2). In this study, the values of EC_SP and EMF in Stage 4 were the lowest compared to other three stages (Figure 4). This most likely occurred because EC_SP and EMF were driven by different indicators at different stages (Cortois et al., 2016). In the Stage 1 and 2, soil factors (i.e., STC, STN, and SM) played dominant roles in influencing EC_SP and EMF. Meanwhile, both plant (PD and PH) and soil factors (STC, STN, SM, and SBD) determined the performance of EC_SP and EMF. in the Stage 4 (Supplementary Figure S2). The site of Stage 3 was associated with the highest values for the SCI and SNI, which may help to explain the highest level of EC_SP and EMF (Figure 5A). Taken together, high ecosystem coupling may show a higher sensitivity response to STC, STN, SM, and STP (Figure 6A). High ecosystem multifunctionality may show a higher sensitivity response to STC, SM, PD, and AGB (Figure 6B). Previous studies had used observational data to indicated that ecosystem functions differ in their sensitivity to grazing pressure (Ren et al., 2018) and restoration practices (Wang et al., 2020b). In this case, the lower ecosystem coupling and multifunctionality in Stage 4 induced by grazing may be caused by several mechanisms. First, heavy foraging activity of yak encouraged unpalatable broad-leaved plants (e.g., *Euphorbia esula* and *Gentiana macrophylla*) to invade. Second, overgrazing by yak leads to alpine meadow degeneration which result in rodent infestation and further grassland degeneration. Furthermore, the deeper and nutrient-poor soil was transferred to the ground level and mixed with topsoil through burrowing activity of rodent, which could contribute to the decrease of SOC and STN in the topsoil (Yu et al., 2017). Also, the latest research found that microbes also mediate the shift in ecosystem multifunctionality (Wang et al., 2021). To further illustrate the mechanism, how soil microbe affects ecosystem coupling and ecosystem multifunctionality in each succession stage needs to be further explored. In addition, our results also suggested that coefficients of correlation between ecosystem coupling and the ecosystem multifunctionality in the earlier stages were higher than those in the later stages (Figure 5B).

**Figure 7:**
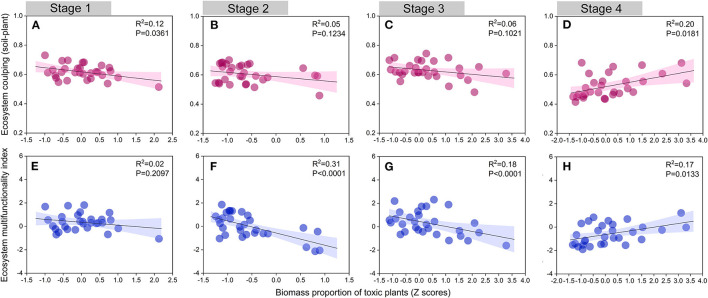
Relationships between ecosystem coupling (soil-plant), ecosystem multifunctionality index, and biomass proportion of toxic weeds (Z scores) in different succession stages. Stage 1, **(A,E)**; Stage 2, **(B,F)**; Stage 3, **(C,G)**; Stage 4, **(D,H)**.

Toxic plants can exhibit self-protective mechanisms in degraded grasslands and promote positive feedback between plants and soil (Zhang et al., 2020). For instance, toxic plants with well-developed root systems can stabilize sand and extract nutrients from the coarser soil (Estapé et al., 2013; Li et al., 2014). Our study also emphasized the critical role of toxic plants during the late stage of succession (Stage 4). We found that both ecosystem coupling and ecosystem multifunctionality showed a significant increase with an increase in the biomass proportion of toxic plants in Stage 4 (Figure 7). In the case of this alpine meadow, we suggest that the expansion of toxic plants is a consequence of their greater suitability form a successional perspective. We thus conclude that the heavily degraded region of this alpine meadow needs toxic plant species, and these species are vital for ecological restoration, as well as for maintaining ecosystem function and coupling.

## Conclusion

Our study quantified the plant succession sequence of alpine meadow induced by grazing with plant functional group approach. The plant succession sequence is from the tall sedge community with erect growth to the short undesirable toxic forbs community with prostrate growth. Ecosystem coupling, ecosystem multifunctionality and their relationships were all the lowest in Stage 4. Our results indicated that the driving factors of ecosystem coupling and ecosystem multifunctionality were soil properties individual in the early succession to plant-soil simultaneously in the late stage. Our results highlight the importance of toxic weeds during the late stage of degraded succession and suggest that the expansion of toxic plants is a consequence of their greater suitability form a successional perspective.

## Data Availability Statement

The original contributions presented in the study are included in the article/Supplementary Material, further inquiries can be directed to the corresponding author.

## Author Contributions

YW, ZW, and FH designed the methodology of this study. YW, ZW, and SC conducted the field work and collected the data. YW, YQ, ZW, and JC analyzed the data. YW, ZJ, QZ, and FH wrote the manuscript. All authors contributed substantially to the revision. All authors contributed to the article and approved the submitted version.

## Funding

This work was supported by the National Natural Science Foundation of China (32161143028 and U21A20242), the Program of National Science and Technology Assistance (KY202002011), the Program for Innovative Research Team of Ministry of Education (IRT17R50), and Lanzhou City's Scientific Research Funding Subsidy to Lanzhou University.

## Conflict of Interest

The authors declare that the research was conducted in the absence of any commercial or financial relationships that could be construed as a potential conflict of interest.

## Publisher's Note

All claims expressed in this article are solely those of the authors and do not necessarily represent those of their affiliated organizations, or those of the publisher, the editors and the reviewers. Any product that may be evaluated in this article, or claim that may be made by its manufacturer, is not guaranteed or endorsed by the publisher.

## Supplementary Material

Supplementary Figure S1 **(a,b)** The cluster dendrogram of sample plot.

Supplementary Figure S2 Correlations among variables of plant, soil properties and ecosystem coupling and multifunctionality in each succession stage, respectively. The value in the square represents the value of the correlation coefficient. ^*^, ^**^, and ^***^ show the significant correlations at 0.05, 0.01, and 0.001 levels. SR, plant species richness; EV, Camargo evenness index; PGI, plant growth index; SCI, soil carbon accumulation index; SNI, soil nutrient index; EMF, ecosystem multifunctionality index; EC_SP, extent of ecosystem coupling (soil-plant); AGB, aboveground biomass; BGB, belowground biomass; PD, plant density; PH, plant height; SBD, soil bulk density; SM, soil moisture; ST, soil temperature; STC, soil total carbon; STN, soil total nitrogen; STP, soil total phosphorus.
