# Supplementary figures and images for: Ecosystem Coupling and Ecosystem Multifunctionality May Evaluate the Plant Succession Induced by Grazing in Alpine Meadow
Source: Front Plant Sci. 2022 Mar 4;13:839920. doi: 10.3389/fpls.2022.839920 (PMC8934431; doi:10.3389/fpls.2022.839920)

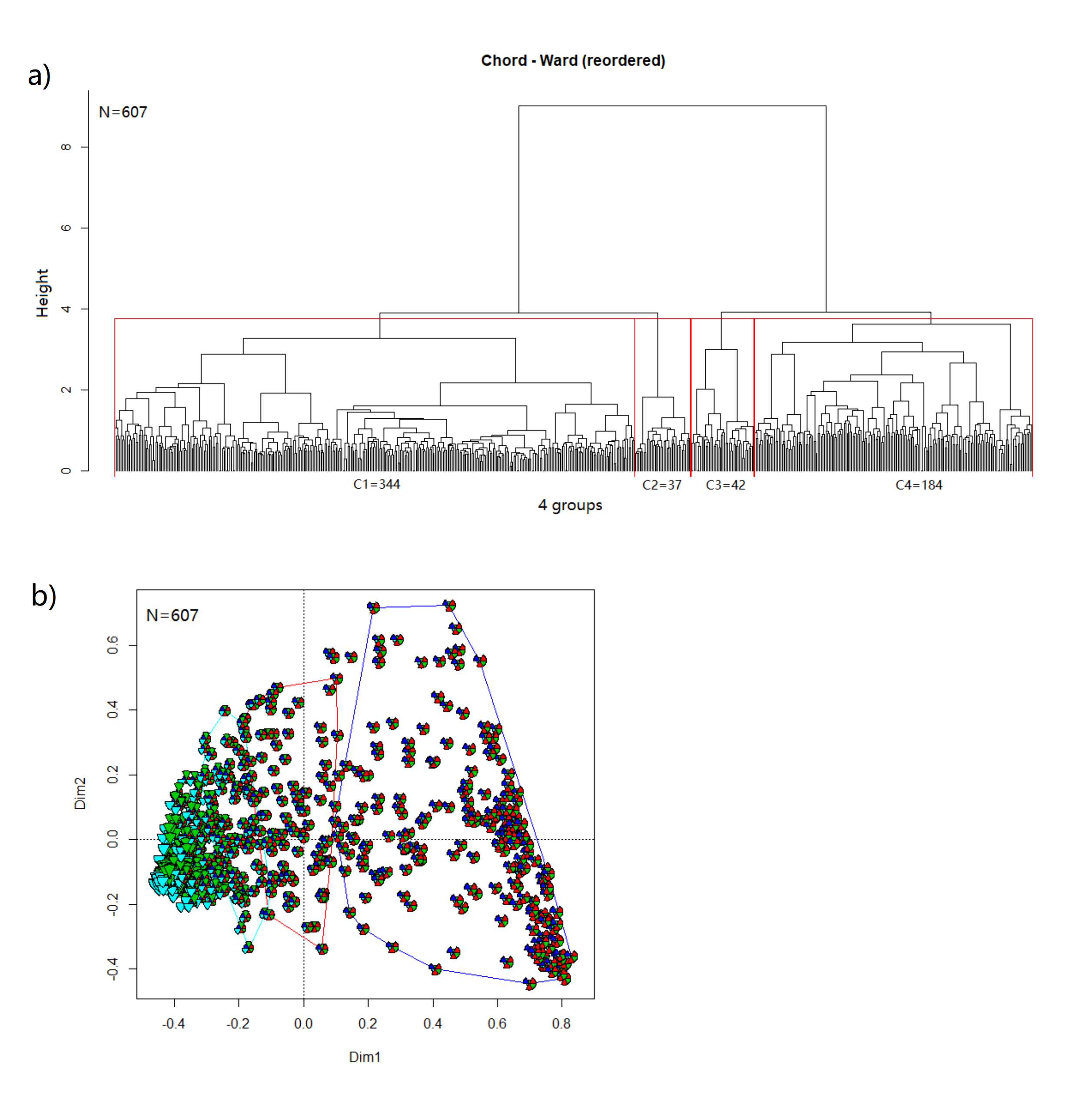

Supplement: Supplementary Figure S1 — (a,b) The cluster dendrogram of sample plot. [file Image_1.TIF]

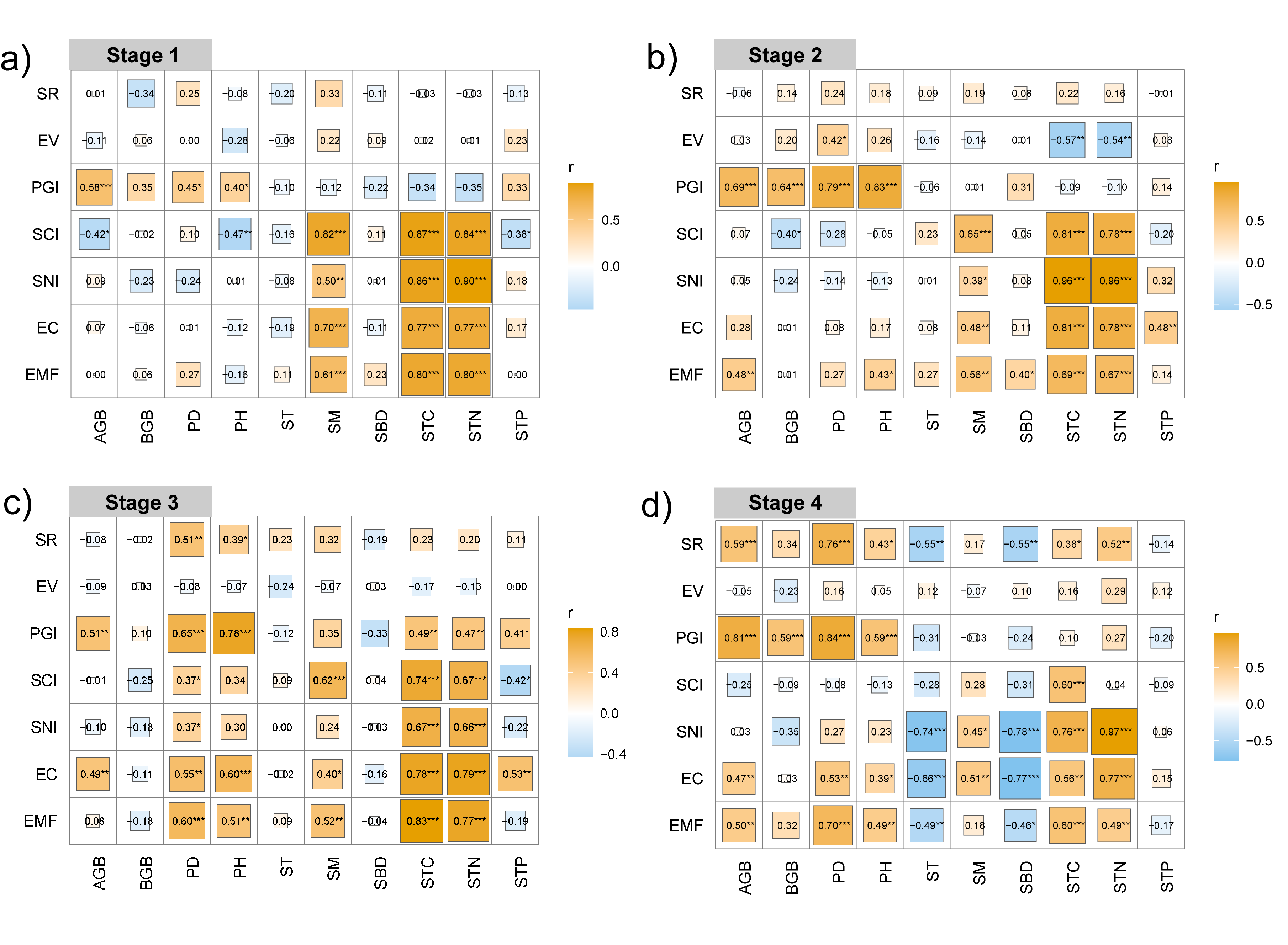

Supplement: Supplementary Figure S2 — Correlations among variables of plant, soil properties and ecosystem coupling and multifunctionality in each succession stage, respectively. The value in the square represents the value of the correlation coefficient. *, **, and *** show the significant correlations at 0.05, 0.01, and 0.001 levels. SR, plant species richness; EV, Camargo evenness index; PGI, plant growth index; SCI, soil carbon accumulation index; SNI, soil nutrient index; EMF, ecosystem multifunctionality index; EC_SP, extent of ecosystem coupling (soil-plant); AGB, aboveground biomass; BGB, belowground biomass; PD, plant density; PH, plant height; SBD, soil bulk density; SM, soil moisture; ST, soil temperature; STC, soil total carbon; STN, soil total nitrogen; STP, soil total phosphorus. [file Image_2.TIF]
